# Subject-Independent Functional Near-Infrared Spectroscopy-Based Brain–Computer Interfaces Based on Convolutional Neural Networks

**DOI:** 10.3389/fnhum.2021.646915

**Published:** 2021-03-12

**Authors:** Jinuk Kwon, Chang-Hwan Im

**Affiliations:** ^1^Department of Biomedical Engineering, Hanyang University, Seoul, South Korea; ^2^Department of Electronic Engineering, Hanyang University, Seoul, South Korea

**Keywords:** brain–computer interface, functional near-infrared spectroscopy, deep learning, convolutional neural network, binary communication

## Abstract

Functional near-infrared spectroscopy (fNIRS) has attracted increasing attention in the field of brain–computer interfaces (BCIs) owing to their advantages such as non-invasiveness, user safety, affordability, and portability. However, fNIRS signals are highly subject-specific and have low test-retest reliability. Therefore, individual calibration sessions need to be employed before each use of fNIRS-based BCI to achieve a sufficiently high performance for practical BCI applications. In this study, we propose a novel deep convolutional neural network (CNN)-based approach for implementing a subject-independent fNIRS-based BCI. A total of 18 participants performed the fNIRS-based BCI experiments, where the main goal of the experiments was to distinguish a mental arithmetic task from an idle state task. Leave-one-subject-out cross-validation was employed to evaluate the average classification accuracy of the proposed subject-independent fNIRS-based BCI. As a result, the average classification accuracy of the proposed method was reported to be 71.20 ± 8.74%, which was higher than the threshold accuracy for effective BCI communication (70%) as well as that obtained using conventional shrinkage linear discriminant analysis (65.74 ± 7.68%). To achieve a classification accuracy comparable to that of the proposed subject-independent fNIRS-based BCI, 24 training trials (of approximately 12 min) were necessary for the traditional subject-dependent fNIRS-based BCI. It is expected that our CNN-based approach would reduce the necessity of long-term individual calibration sessions, thereby enhancing the practicality of fNIRS-based BCIs significantly.

## Introduction

Brain–computer interfaces (BCIs) have been developed to decode a user's intention from their neural signals with the ultimate goal of providing non-muscular communication channels to those who experience difficulties communicating with the external environment (Wolpaw et al., 2002; Daly and Wolpaw, 2008). Various neuroimaging modalities such as electroencephalography (EEG), magnetoencephalography, and functional magnetic resonance imaging have been employed to implement BCIs (Mellinger et al., 2007; Sitaram et al., 2007; Hwang et al., 2013). Recently, functional near-infrared spectroscopy (fNIRS), which is also one of the representative brain-imaging modalities, has attracted increasing attention owing to its advantages, including non-invasiveness, affordability, low susceptibility to noise, and portability (Naseer and Hong, 2015; Shin et al., 2017a). fNIRS is an optical brain-imaging technology used to record hemodynamic responses of the brain using near-infrared-range light of wavelength 600–1,000 nm. fNIRS can measure oxy- and deoxy-hemoglobin concentration changes (ΔHbO and ΔHbR) while an individual performs specific mental tasks such as mental arithmetic (MA), motor imagery (MI), mental singing, and imagining of object rotation. During these mental tasks, increased cerebral blood flow caused by neural activities leads to an increase and decrease in ΔHbO and ΔHbR, respectively, which have been utilized to implement fNIRS-based BCIs (Ferrari and Quaresima, 2012; Schudlo and Chau, 2015). Previous studies (Coyle et al., 2007; Naseer and Hong, 2013; Hong et al., 2020) have reported that the performance of fNIRS-based BCI is high enough to be applied to practical binary communication systems that require a threshold classification accuracy of at least 70% (Vidaurre and Blankertz, 2010).

Recently, many researchers have proposed new approaches to improve the performance of fNIRS-based BCIs. For example, recent studies have reported significant improvements in the classification accuracy of fNIRS-based BCIs by employing high-density multi-distance fNIRS devices (Shin et al., 2017a) and using ensemble classifiers based on bootstrap aggregation Shin and Im (2020). von Lühmann et al. (2020) proposed a general linear model-based preprocessing method to improve the classification accuracy of fNIRS-based BCI. The combination of fNIRS with other brain-imaging modalities also demonstrated a potential to improve the classification accuracy of the BCI system (Fazli et al., 2012; Shin et al., 2018b). Recently, Kwon and Im (2020) demonstrated that photobiomodulation before a BCI experiment could enhance the overall classification accuracy of fNIRS-based BCIs. Besides, a number of studies have attempted to improve the information transfer rate (ITR) of fNIRS-based BCI by increasing the number of commands (i.e., mental tasks) (Khan et al., 2014; Hong and Khan, 2017; Shin et al., 2018a). In addition, researchers have also been interested in implementing portable BCI systems with a small number of sensors while preserving the overall BCI performance to elevate their practical applicability (Kazuki and Tsunashima, 2014; Shin et al., 2017b; Kwon et al., 2020a).

Although fNIRS-based BCI technology has advanced considerably, it is still challenging to use fNIRS-based BCIs in real-world applications because neural signals generally exhibit high inter-subject variability and non-stationarity. Moreover, because fNIRS signals are readily affected by a user's mental state, such as cognitive load and fatigue, they can change during the course of same-day experiments (Holper et al., 2012; Hu et al., 2013). Therefore, individual training sessions need to be performed before each usage of the BCI system to acquire high-performance BCI systems. However, such relatively long calibration sessions to obtain enough training data degrade their practicality and sometimes cause user fatigue even before using the BCI system. Various strategies have been proposed to reduce the necessity of such long-term calibration sessions in the field of EEG-based BCIs (Fazli et al., 2009; Wang et al., 2015; Yuan et al., 2015; Jayaram et al., 2016; Waytowich et al., 2016; Joadder et al., 2019; Xu et al., 2020). Recently, Kwon et al. (2020b) proposed a subject-independent EEG-based BCI framework based on deep convolutional neural networks (CNNs), which does not require any calibration sessions, with a fairly high classification accuracy. However, to the best of our knowledge, no previous study has successfully implemented a deep CNN-based subject-independent fNIRS-based BCI that outperforms conventional machine-learning-based subject-independent fNIRS-based BCIs.

In this study, we proposed a novel CNN-based deep-learning approach for subject-independent fNIRS-based BCIs. fNIRS signals were recorded using a portable fNIRS recording system that covers the prefrontal cortex while the participants were performing MA and idle state (IS) tasks. The leave-one-subject-out cross-validation (LOSO-CV) strategy was employed to evaluate the performance of the proposed method. The resultant classification accuracy was then compared with the threshold accuracy for effective binary BCIs (70%) and the classification accuracy was achieved using the conventional machine learning method, which has been widely employed for fNIRS-based BCIs. To the best of our knowledge, this is the first study that has applied a deep learning approach to subject-independent fNIRS-based mental imagery BCIs.

## Materials and Methods

### Dataset

**Figure 1:**
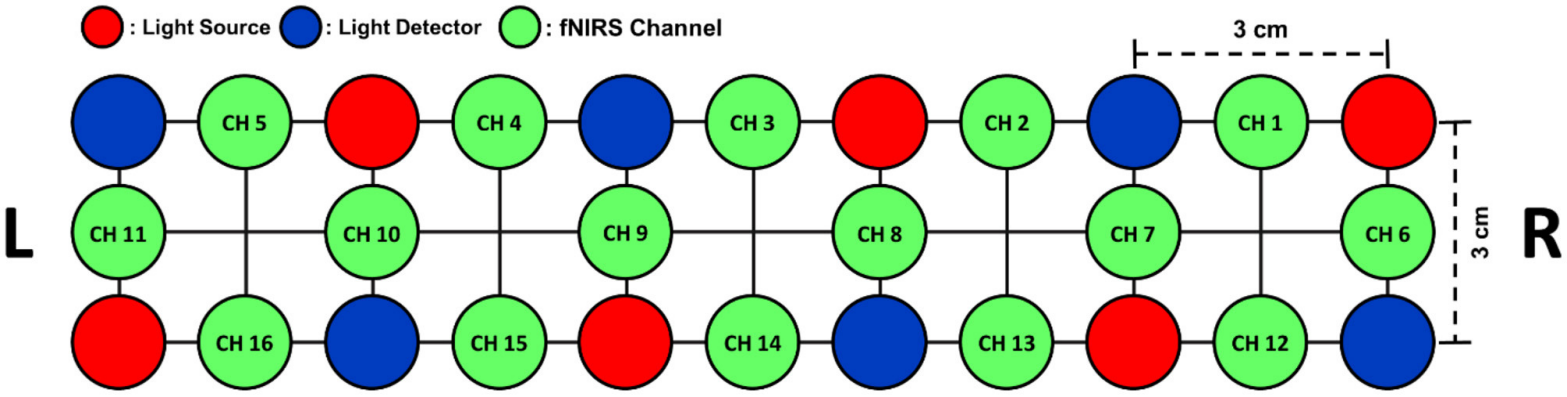
The arrangement of six light emitters (red) and six light detectors (blue) on the forehead over the prefrontal area. A total of 16 fNIRS channels (green) were formed by the pairs of neighboring light emitters and detectors with a distance of 3 cm between them.

In this study, a part of an fNIRS dataset collected in our previous study (Shin et al., 2018b) was used to evaluate the proposed method. The original dataset consisted of 21-channel EEG data and 16-channel fNIRS data, which were recorded from 18 healthy adult participants (10 males and 8 females, 23.8 ± 2.5 years). From the original dataset, only the fNIRS data measured during the MA and IS tasks at all 16 prefrontal NIRS channels were selectively used in this study. A commercial NIRS recording system (LIGHTNIRS; Shimadzu Corp.; Kyoto, Japan) was used to record fNIRS signals at a sampling rate of 13.3 Hz. The arrangement of the fNIRS channels is shown in Figure 1.

### Experiment Paradigm

**Figure 2:**
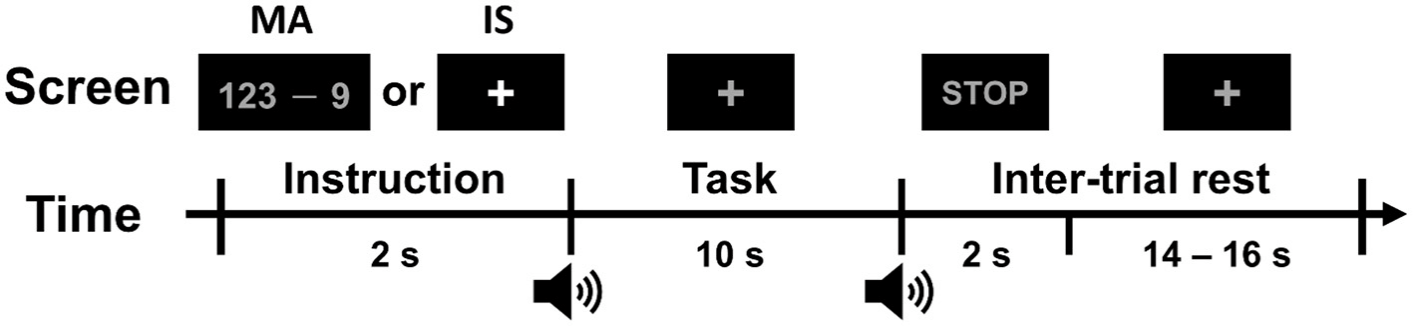
Timing sequence of a single trial. Each trial consisted of an introduction period of 2 s, a task period of 10 s, and an inter-trial rest period of 16–18 s. During the introduction period, the task to be performed was displayed at the center of the monitor. After a short beep, the participants were asked to perform the designated task while looking at a fixation cross. When a STOP sign was displayed with a second short beep, the participants stopped performing the task and relaxed during the inter-trial rest period.

The timing sequence of a single trial is shown in Figure 2. Each task trial consisted of an instruction (2 s), task (10 s), and inter-trial rest (a randomized interval of 16–18 s). During the instruction period, a specific task to be performed during the task period was displayed at the center of the monitor. The participants were provided with either a mathematical expression showing a “random three-digit number minus a one-digit number between 6 and 9 (e.g., 123–9)” for the MA task or a fixation cross for the IS task. During the task period, the participants were asked to perform either MA or IS tasks as instructed. During the MA task, the participants had to repetitively subtract the designated one-digit number from the result of the former calculation as quickly as possible (e.g., 123–9 = 114, 114–9 = 105, 105–9 = 96, …), until the stop sign was presented. During the IS task, the participants stayed relaxed without performing any mental imagery task. The MA and IS tasks were performed 30 times each.

### Preprocessing

MATLAB 2018b (MathWorks; Natick, MA, USA) was used to analyze the recorded fNIRS data, when functions implemented in the BBCI toolbox^1^ were employed. The raw optical densities (ODs) were converted to ΔHbR and ΔHbO using the following formula (Matcher et al., 1995):

$$\begin{array}{l} \left(\begin{array}{c} \Delta \text{HbR}\\ \Delta \text{HbO} \end{array}\right)=\left(\begin{array}{ccc} 1.8545 & -0.2394 & -1.0947\\ -1.4887 & 0.5970 & 1.4847 \end{array}\right)\left(\begin{array}{c} \Delta \text{O}{\text{D}}_{780}\\ \Delta \text{O}{\text{D}}_{805}\\ \Delta OD_{830} \end{array}\right)\;\left(\text{mM }\cdot \text{ cm}\right), \end{array}$$

swhere ΔOD represents the optical density changes at wavelengths of 780, 805, and 830 nm. The converted ΔHbR and ΔHbO values were band-pass filtered at 0.01–0.09 Hz using a 6th-order Butterworth zero-phase filter to remove physiological noise. fNIRS data were then segmented into epochs from 0 to 15 s considering the hemodynamic delay of the order of several seconds (Naseer and Hong, 2013). Baseline correction was performed by subtracting the temporal mean value within the (−1 s, 0 s) interval from each fNIRS epoch.

### Performance Evaluation

#### Shrinkage Linear Discriminant Analysis

A shrinkage linear discriminant analysis (sLDA), which is a combination of linear discriminant analysis (LDA) and a shrinkage tool, was employed as the representative conventional classification method as it has been widely employed in recent fNIRS-based BCI studies owing to its high classification performance (Shin et al., 2017a, 2018b). This method is known to be particularly useful for improving the estimation of covariance matrices in situations where the number of training samples is small compared to the number of features. The feature vectors to train the sLDA were constructed using the temporal mean amplitudes of fNIRS data within multiple windows of 0–5, 5–10, and 10–15 s for each epoch. As a result, the dimension of fNIRS feature vectors was 96 (= 16 channels × 2 fNIRS chromophores × 3 intervals).

#### Proposed Deep Learning Approach

We proposed a one-dimensional CNN-based deep-learning approach for subject-independent fNIRS-based BCI. The detailed network architecture is listed in Table 1. The proposed model consisted of an input layer, two 1-dimensional convolutional layers, and a single fully connected layer. The input layer had a dimension of 201 (time samples) × 32 (= 16 channels × 2 chromophores), followed by two convolutional layers with 32 filters. The kernel sizes of the two layers were set to 13 and 6, and the stride sizes of the two layers were set to 9 and 4. The flattened output of the last convolutional layer, which had the dimension of 128, was fed into the fully connected layer, followed by the Softmax activation function. Consequently, the output of the proposed method had a dimension of two, corresponding to the number of tasks to be classified. The normalization and dropout layers were added after the input layer and the two convolutional layers to improve the generalization performance and training speed of the networks (Ravi et al., 2020). An evolving normalization-activation layer (EvoNorm) (Liu et al., 2020) was employed as the normalization layer, and the dropout probability was set to 0.5. The weights of the layers were initialized using a He-Normal initializer.

**Table 1:** The architecture of the deep-learning model based on 1-dimensional CNN.

**Layer**	**Number of filters**	**Kernel size**	**Normalization, dropout, activation layer**	**Output shape**	**Options**
Input			EvoNorm Dropout (*p* = 0.5)	(201, 32)	
1D Conv	32	13	EvoNorm Dropout (*p* = 0.5)	(21, 32)	Stride = 9
					Padding = Valid
1D Conv	32	6	EvoNorm Dropout (*p* = 0.5)	(4, 32)	Stride = 4
					Padding = Valid
Flatten				(128)	
Dense	128 × 2		Softmax	(2)	

#### Ensemble of Regularized LDA

Recently, Shin and Im (2020) demonstrated that ensemble of weak classifiers resulted in a better classification accuracy than that of a single strong classifier. Based on this work, the ensemble of regularized LDA based on bootstrap aggregating (Bagging) algorithm was employed to validate the performance of subject-independent fNIRS-based BCI. The Bagging algorithm creates multiple training sets by sampling with replacement, then builds weak classifiers using each training set. The final classification result is decided by a majority vote of results from weak classifiers. In this study, the ensemble classifier was implemented using the MATLAB “fitcensemble” function. According to the previous study (Shin and Im, 2020), the number of weak classifiers, fraction of training set to resample, and gamma value for regularized LDA were set to 50, 100%, and 0.1, respectively. The feature vectors of training sets were set to be the same as those used to train sLDA.

#### EEGNet

Lawhern et al. (2018) introduced a compact CNN-based deep-learning architecture (EEGNet) that contains a small number of training parameters but showed robust classification performance in various EEG-based BCI paradigms such as P300, error-related negativity, movement-related cortical potential, and sensory-motor rhythm during MI. In this study EEGNet was employed as a conventional CNN-based classification method to verify the performance of the proposed method. EEGNet consists of an input layer, three 2-dimensional convolutional layers of temporal, spatial, and separable layers, and a single fully connected layer as listed in Table 2. The input layer had a dimension of 32 (= 16 channels × 2 chromophores) × 201 (time samples) × 1, followed by a 2-dimenssional temporal convolutional layer with *F*_1_ filters. The kernel size of the temporal convolutional layer was set to (1, 6), chosen to be half the sampling rate of the data. The spatial convolutional layer had *D* × *F*_1_ filters with the kernel size of (32, 1), and the separable convolutional layer had *F*_2_ filters with the kernel size of (2, 1). Each convolutional layer was followed by a Batch Normalization layer (BatchNorm) and a linear or exponential linear unit activation layer (ELU). Two average pooling layers were located after spatial and separable layers to reduce the size of feature maps, with the kernel sizes of (1, 4) and (1, 8), respectively. In this study, all the hyper parameters were determined based on the previous studies (Lawhern et al., 2018). *F*_1_, *F*_1_, and *D* were set to 8, 16, and 2, respectively, and the kernel sizes of each convolutional layer were set considering the sampling rate of the fNIRS device.

**Table 2:** The architecture of EEGNet.

**Layer**	**Number of filters**	**Kernel size**	**Normalization, Dropout, activation layer**	**Output shape**	**Options**
Input				(32, 201, 1)	
2D Conv	*F*_1_	(1, 6)	BatchNorm	(32, 201, *F*_1_)	Padding = same
2D Depthwise Conv	*D* × *F*_1_	(32, 1)	BatchNorm ELU	(1, 201, *D* × *F*_1_)	Padding = valid Depth = *D* Max norm = 1
2D Average Pooling		(1, 4)	Dropout (*p* = 0.25)	(1, 50, *D* × *F*_1_)	
2D Separable Conv	*F*_2_	(1, 2)	BatchNorm ELU	(1, 50, *F*_2_)	Padding = same
2D Average Pooling		(1, 8)	Dropout (*p* = 0.25)	(1, 6, *F*_2_)	
Flatten				1 × 6 × *F*_2_	
Dense	(6 × *F*_2_) × 2		Softmax	2	

*F_1_, F_2_, and D were set to 8, 16, and 2, respectively*.

#### Training Details

All the training and simulation processes were run on a desktop computer with a 12-core Ryzen 9 3900x processor, 64 GB memory, and an NVIDIA RTX 2080Ti GPU, using Keras (https://keras.io) with a Tensorflow backend, which is an open-source library for deep learning. Ten percent of the training data was split as the validation set, and an early stopping technique with a patience of 20 was used to avoid over-fitting with a batch size of 100. The hyper-parameters were empirically determined, and the random seed was set to 0. The pre-processed fNIRS data were fed into the proposed network after *z*-score normalization over the time axis to compensate for intrinsic amplitude differences among participants (Erkan and Akbaba, 2018). The network was trained to minimize the categorical cross-entropy loss function using the Adamax optimizer (Kingma and Ba, 2014; Vani and Rao, 2019) with a learning rate of 0.0005, decay of 5 × 10^−8^.

#### Leave-One-Subject-Out Cross-Validation

A leave-one-subject-out cross-validation (LOSO-CV) strategy was employed to evaluate the performance of subject-independent fNIRS-based BCIs. In LOSO-CV, all the datasets except for a test participant—that is, the dataset of 1,020 samples (= 17 participants × 30 trials × 2 classes)—were used to train the classifier, and then data from the test participant (30 trials × 2 classes = 60 samples) were classified to evaluate the performance of the trained classifier. For example, when participant #1 was a test participant, the classification model for the participant #1 was trained using the data of the other 17 participants (participants #2 to #18). Then, the accuracy of the trained model was evaluated by applying the participant #1's data that were not used for the training to the trained model. This process was repeated until all participants' data were tested.

#### Pseudo-Online Simulation of Subject-Dependent fNIRS-Based BCI

A pseudo-online simulation of subject-dependent fNIRS-based BCI was performed to investigate how many training trials were required to achieve a classification accuracy higher than that of subject-independent fNIRS-based BCI. The dataset of each participant was split into training data and test data. For each task, the first *N* trials and the remaining (30 - *N*) trials were used as the training and test datasets, respectively. sLDA was employed as the classifier (Shin et al., 2017a) for this subject-dependent fNIRS-based BCI, and the classification accuracy was evaluated for different sizes (*N*) of training datasets to investigate how many training trials each participant should undergo before using the fNIRS-based BCI. It should be noted that data from other participants were not utilized to train the classifier.

## Results

**Figure 3:**
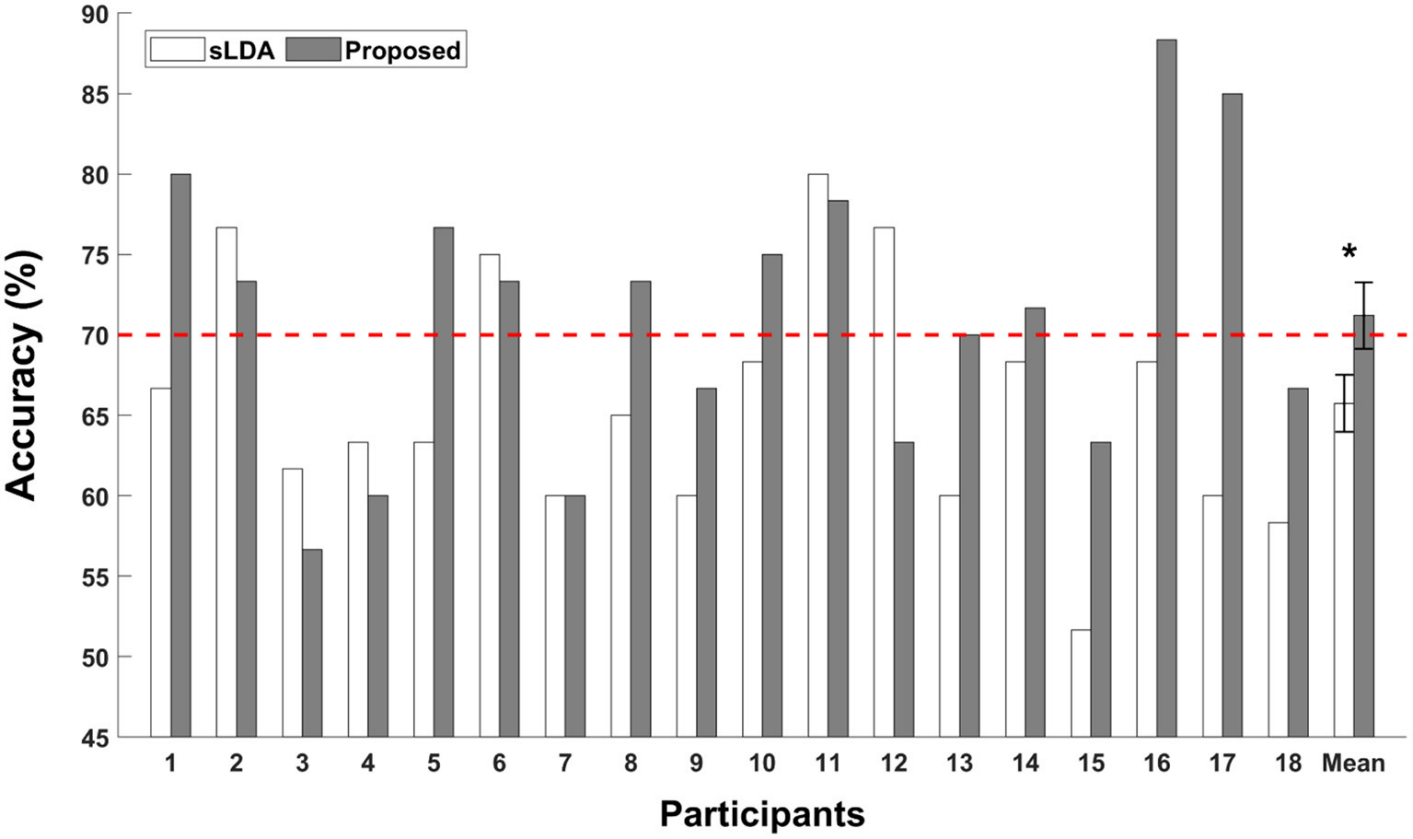
Individual classification accuracies of the subject-independent fNIRS-based BCI. White and gray bars indicate the classification accuracies obtained using the shrinkage linear discriminant analysis (sLDA) classifier and the proposed method. The red horizontal dashed line indicates the effective BCI threshold level (70.0%). Error bars represent the standard errors. The grand average classification accuracies were 65.74 ± 7.68% and 71.20 ± 8.74% (mean ± standard deviation) for the sLDA and the proposed method, respectively. The asterisk (*) represents *p* < 0.05 (Wilcoxon signed rank test).

The binary classification accuracies of individual participants are shown in Figure 3. The white and gray bars represent the classification accuracies of subject-independent fNIRS-based BCIs implemented using sLDA and the proposed CNN-based methods, respectively. The error bars represent the standard errors. The red dotted horizontal line denotes the threshold accuracy for the effective binary BCI (70%). The average classification accuracy of the proposed method was reported to be 71.20 ± 8.74% (mean ± standard deviation), which was higher than that obtained using the conventional sLDA (65.74 ± 7.68%) as well as the threshold accuracy for effective binary BCI communications (70%). The Wilcoxon signed rank sum test was conducted to statistically compare the difference in the classification accuracies, and statistically significant improvement of classification accuracy was observed for the proposed method (*p* < 0.05).

**Figure 4:**
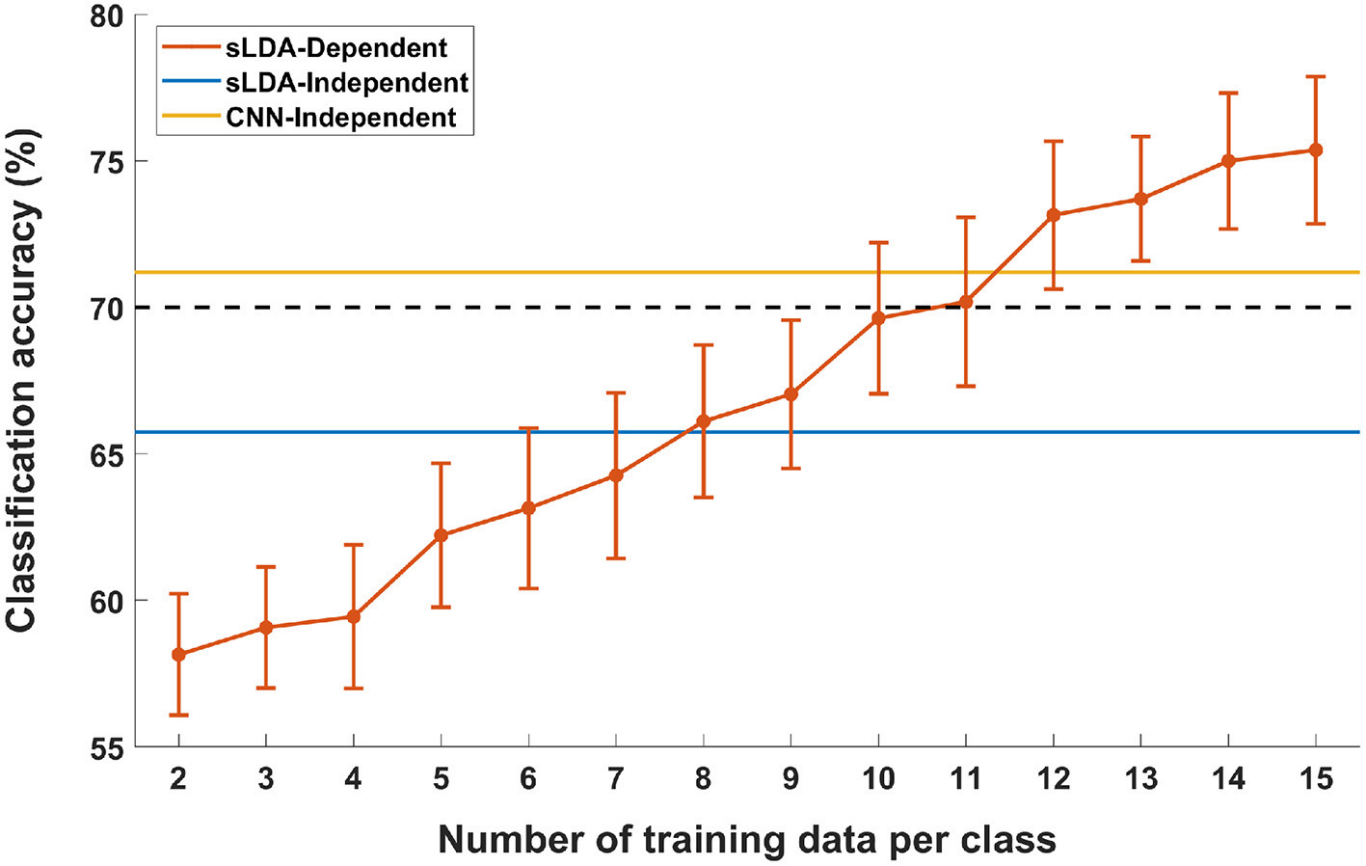
Comparison of MA vs. IS classification accuracies of subject-independent (sLDA-independent and CNN-independent) and subject-dependent (sLDA-dependent) scenarios as a function of the number of individual training data. Vertical lines indicate the standard errors. The black horizontal dashed line represents the threshold accuracy of the effective BCI application (70.0%).

Figure 4 shows the results of the pseudo-online simulation of subject-dependent fNIRS-based BCI (denoted by “sLDA-Dependent” in the figure) with respect to different numbers of training data per class. The two horizontal lines denoted by “sLDA-independent” and “CNN-independent” represent the average accuracies of subject-independent fNIRS-based BCIs achieved using sLDA (65.74%) and CNN (71.20%), respectively. The black dotted line represents the threshold accuracy for an effective binary BCI (70%). It can be seen from the figure that the overall classification accuracy of the subject-dependent BCI increased as the number of training data increased. Notably, at least 12 training data per class were required to realize a subject-dependent fNIRS-based BCI with better performance than the subject-independent fNIRS-based BCI implemented using the proposed CNN-based method. This implies that an approximately 12 m-long training session may not be necessary before using the fNIRS-based BCI if the proposed subject-independent fNIRS-based BCI is employed.

**Figure 5:**
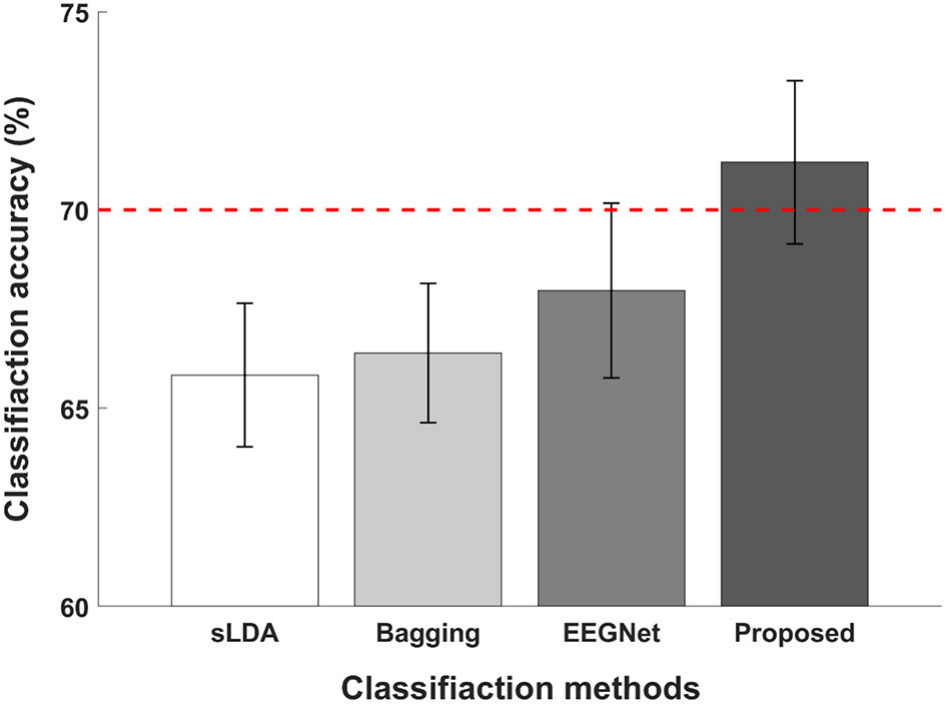
Comparison of average classification accuracies of sLDA, ensemble of regularized LDA (denoted by “Bagging”), EEGNet, and the proposed method. The red horizontal dashed line indicates the effective BCI threshold level (70.0%) and error bars represent the standard errors. The average classification accuracies were 65.74 ± 7.68, 66.39 ± 7.44, 67.96 ± 9.35, and 71.20 ± 8.74% for sLDA, Bagging, EEGNet, and the proposed method, respectively.

Figure 5 illustrates the average classification accuracies of subject-independent fNIRS-based BCI evaluated using different classification methods. The red dotted horizontal line denotes the threshold accuracy for the effective binary BCI (70%) and the error bars represent the standard errors. The average classification accuracies evaluated using sLDA, ensemble of regularized LDA (denoted by “Bagging” in the figure), EEGNet, and proposed CNN-based methods were reported to be 65.74 ± 7.68%, 66.39 ± 7.44%, 67.96 ± 9.35%, and 71.20 ± 8.74%. Among all classification methods, only the proposed CNN-based method achieved higher classification accuracy than the threshold accuracy for effective binary BCI communications.

## Discussion

In this study, we investigated the feasibility of implementing a subject-independent fNIRS-based BCI using a deep learning-based approach. We proposed a novel deep-learning-based model architecture based on a CNN to effectively differentiate the two mental tasks, MA and IS. fNIRS signals were recorded from 16 sites covering the prefrontal cortex while participants performed either MA or IS task. The classification accuracy obtained using the proposed CNN-based method was reported to be 71.20 ± 8.74%, which was not only higher than the threshold accuracy for effective BCI communication, but also higher than that obtained using the conventional sLDA method. Our experimental results demonstrated that our deep-learning-based approach has great potential to be adopted to establish a zero-training fNIRS-based BCI that could significantly enhance the practicality of fNIRS-based BCIs.

We believe that the improvement in the overall BCI performance stemmed from the synergetic effect of three factors employed to construct the proposed CNN-based model architecture. First of all, the CNN layer had high automatic feature extraction ability compared to that of the conventional feature extraction method (Shaheen et al., 2016). Additionally, construction of an appropriate structure of fully-connected layers is also an important factor. The performances of subject-independent fNIRS-based BCIs using various fully-connected layers with different structures are listed in Supplementary Table 1. Finally, to improve the generalization performance, we adopted EvoNorm, a recently introduced normalization-activation layer (Liu et al., 2020), instead of a batch normalization layer followed by the ReLU activation layer, which is a widely-used approach in deep learning. The classification accuracy evaluated using the EvoNorm (71.20%) was significantly higher than that obtained using the batch normalization and the ReLU activation layers (68.43%, *p* < 0.05, Wilcoxon signed rank test).

A previous study on the implementation of a subject-independent EEG-based BCI (Kwon et al., 2020b) reported the average classification accuracy of 74.15% in the two-class MI task classification problem. Since the modalities and paradigms of the previous study and this study are quite different with each other, direct comparison of BCI performance may not be meaningful; however, some important clues that can be employed in our future studies could be found in the previous study. In Kwon et al.'s study, EEG data were recorded from a total of 54 participants, which was almost three times more than the number of participants participated in our experiments. The authors of the previous study (Kwon et al., 2020b) demonstrated that a deep neural network model trained with a larger number of training data could result in a better classification accuracy and reduce the differences in BCI performance among participants. Thus, it may be a promising topic to investigate whether the performance of subject-independent fNIRS-based BCI based on our proposed CNN model could be further enhanced by increasing the size of the fNIRS dataset through additional experiments with a larger number of participants. The application of data augmentation techniques (Luo and Lu, 2018) or the employment of open-access datasets (Shin et al., 2018c) could also be promising options to increase the training data without additional experiments. After increasing the number of training data large enough to improve the overall BCI performance and investigating more appropriate deep learning structures, we will implement a real-time fNIRS-based BCI communication system that does not require any training session.

Current trends in BCI research are moving toward a hybrid BCI approach that combines more than two neuroimaging modalities to improve BCI performance. Among the various possible hybrid BCIs, a hybrid fNIRS-EEG BCI has been widely studied and has demonstrated the potential to increase the overall performance of BCIs—particularly compared to that of unimodal BCIs in terms of both classification accuracy and ITR (Hong and Khan, 2017; Shin et al., 2018b). Because Kwon et al. (2020b) recently demonstrated the feasibility of implementing a subject-independent EEG-based BCI using CNN, it is expected that a subject-independent hybrid fNIRS-EEG BCI could also be implemented by incorporating our proposed CNN model for fNIRS-based BCI with Kwon et al.'s CNN model for EEG-based BCI.

In this study, the proposed CNN-based model was trained using the data from different participants, excluding the data from the test participant. Although this study focused only on the feasibility of implementing subject-independent BCIs, the classification accuracy could be further improved by adopting a fine-tuning technique (Bengio, 2012; Anderson et al., 2016) with a small portion of the test subject's data. The fine-tuning technique has shown promising results, particularly when a deep learning model needs to be trained using only a small number of datasets. If this “few-training” approach could dramatically increase the classification accuracy of the fNIRS-based BCI, then just a few minute training sessions before the use of the BCI system would be manageable. This would be one of the promising areas we would like to investigate in our future studies.

In this study, the proposed CNN-based approach has demonstrated its potential to be used to implement a practical subject-independent fNIRS-based BCI; however, we believe that there is still room for improvement in future studies. First, the proposed deep learning approach is based on CNNs, but there are other promising neural network models—such as long short-term memory (LSTM)—which are known to be particularly effective for dealing with time-series data. Asgher et al. (2020) reported that the deep learning framework based on LSTM outperformed conventional machine learning and CNN-based algorithms in the assessment of cognitive and mental workload using fNIRS. Therefore, it would be worthwhile to compare the performance of various deep learning approaches in the implementation of subject-independent fNIRS-based BCI. In addition, we used raw fNIRS data without any particular feature extraction method except for band-pass filtering and Z-score normalization as the input tensor of the CNN model. Furthermore, investigating the feasibility of new forms of input tensors (e.g., adjacency matrix of functional connectivity network) to implement a subject-independent fNIRS-based BCI would be an interesting research topic.

## Data Availability Statement

Publicly available datasets were analyzed in this study. This data can be found here: https://doi.org/10.6084/m9.figshare.9198932.v1.

## Ethics Statement

The studies involving human participants were reviewed and approved by Institutional Review Board Committee of Hanyang University. The patients/participants provided their written informed consent to participate in this study.

## Author Contributions

JK planned the study and analyzed the data. C-HI supervised the study. Both authors wrote and reviewed the manuscript.

## Conflict of Interest

The authors declare that the research was conducted in the absence of any commercial or financial relationships that could be construed as a potential conflict of interest.

## Footnotes

^1^https://github.com/bbci/bbci_public
