# Supplementary material for: Subject-Independent Functional Near-Infrared Spectroscopy-Based Brain–Computer Interfaces Based on Convolutional Neural Networks
Source: Front Hum Neurosci. 2021 Mar 12;15:646915. doi: 10.3389/fnhum.2021.646915 (PMC7994252; doi:10.3389/fnhum.2021.646915)
Supplement: Supplementary file 1 [file Data_Sheet_1.PDF]

Supplementary Material

Supplementary Table 1.

The average classification accuracies of subject-independent fNIRS-based BCI with respect to different fully connected layer structures

Model Structure	Input layer<br>1D Conv layers<br>Flatten (Output Shape = 128)						
	Dense (2)<br>Softmax	Dense (16)<br>Dense (2)<br>Softmax	Dense (32)<br>Dense (2)<br>Softmax	Dense (64)<br>Dense (2)<br>Softmax	Dense (32)<br>Dropout<br>Dense (2)<br>Softmax	Dense (96)<br>Dense (32)<br>Dense (2)<br>Softmax	Dense (32)<br>Dense (16)<br>Dense (2)<br>Softmax
Average Classification Accuracy	71.20%	68.06%	69.63%	69.08%	68.24%	69.17	69.17
